# Partnering With Residents on the Redesign of the Internal Medicine Resident Self-Evaluation Form

**DOI:** 10.7759/cureus.33304

**Published:** 2023-01-03

**Authors:** Carolina Borz-Baba, Mohamed Elgamal, Olayinka Agboola, Jackeline P Vajta Gomez, Amritha Alapati, Shawnette Alston

**Affiliations:** 1 Internal Medicine, Saint Mary's Hospital, Waterbury, USA; 2 Medicine, Saint Mary's Hospital, Waterbury, USA

**Keywords:** resident participation, redesign, program, internal medicine, resident self-evaluation

## Abstract

Introduction: The positive impact of resident-driven synthesis of assessment data has been associated with increased intrinsic motivation to learn and create an individualized strategy to improve performance. The objective of the study was to incorporate residents’ recommendations for restructuring the self-assessment metric into a tool that will promote a well-organized and effective self-improvement plan.

Materials and methods: Residents and faculty collaborated on pre- and post-intervention questionnaires to assess the barriers to the timely completion of the current self-evaluation form and gather information on the tool's ability to stimulate the formation of concrete goals. The residents were also invited to provide their recommendations on the structure of the new tool and the educational domains that were assessed by the tool. The post-survey also evaluated the capacity of the proposed tool to guide residents in establishing specific goals.

Results: The new form is concise and more precise in assisting the learner in developing short-term and long-term goals and the strategies and resources to achieve them.

Discussion: Collaborating with the learners created an opportunity to address the faculty's and residents' most important concerns about the effectiveness of the metric.

Conclusion: In a learner-centered model, resident participation is critical in designing/redesigning a practical self-assessment tool for residents in Internal Medicine.

## Introduction

The practice-based learning and improvement competency incorporates resident self-assessment as a fundamental tool to assist self-directed learning. The Accreditation Council for Graduate Medical Education (ACGME)’s program requirements emphasize the importance of residents’ self-reflection on the progress made in their professional growth and the establishment of individualized learning plans (ILP) [1]. The structure of the self-assessment metric is naturally affected by the variability that exists across programs regarding their educational objectives. Still, it reflects the current common milestone recommendations proposed by the ACGME for all programs to minimize inconsistencies among goals [2].

Evidence-based literature on the self-evaluation tool for residents in Internal Medicine (IM) programs is scarce, which prevents us from commenting on the best approach to formulating the metric. As suggested by its description, self-assessment is a method that intimates the benefits of direct learner participation in its originating process. The focus of the study was to incorporate the residents' suggestions on the content and extent of the form and to include them in creating rubrics and reviewing the final version.

## Materials and methods

Participants in this study were residents of a Yale-affiliated community-based IM Residency Program. Residents from each of the three training years were invited to restructure the self-assessment process. A literature review was conducted to determine the most effective structure of the new metric. The resident-faculty group decided to explore the residents’ concerns about the previous format and content. The faculty’s assessment of the current form was that it was completed superficially and submitted at the last minute. The major residents' critiques were that the current form did not provide guidance to help the learner establish well-organized and specific goals and did not suggest strategies or available resources to help the learners achieve their objectives. An anonymous paper-based pre-intervention survey was distributed to 30 IM trainees in July 2021. The survey explicitly inquired about the impact of the length of the evaluation form on the timeliness of completion and the effectiveness of the evaluation in assisting learners with establishing short-term and long-term goals and corresponding strategies to achieve those objectives. We formulated the new metric, and we assessed the learners’ satisfaction with a post-intervention questionnaire. The surveys used a five-point Likert scale grading system (strongly agree, agree, neutral, disagree, and strongly disagree). Answers with strongly agree and agree were grouped and denoted as 1, while answers with neutral, disagree, and strongly disagree were denoted as 0. The statistical analysis method used chi-square and Fisher's exact test as applicable. A P-value <0.05 was considered significant.

## Results

**Figure 1:**
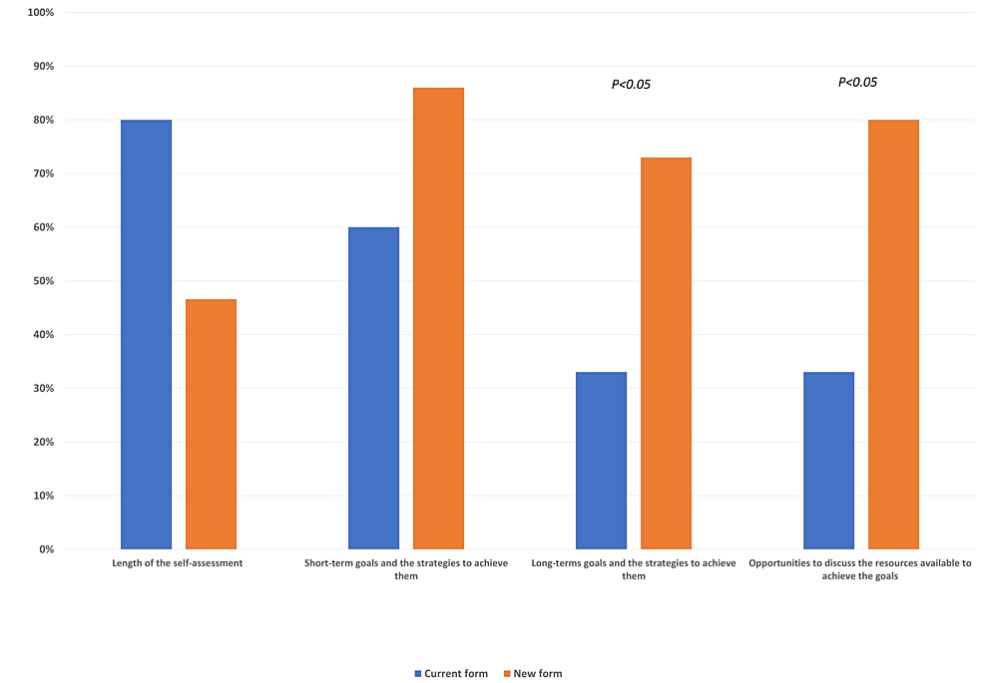
Pre- and post-intervention survey results.

Of the 30 residents surveyed, 15 completed the pre- and post-intervention questionnaires. The results are depicted in Figure 1. Approximately half of the respondents (46.6%) agreed that the current format was lengthy and prevented them from completing it on time. In the post-intervention survey, 80% of the residents found the new form less extensive. Regarding the utility of the metric to establish residents' short-term goals and the strategies to achieve them, 86.67% agreed that the new format enables them to focus on instituting specific aims compared with 60% for the current form. Concerning the long-term goals and strategies, 73.33% of trainees agreed that the new format could better satisfy that objective, contrasting with 33.33% for the previous arrangement (P < 0.05). Lastly, 80% of residents agreed that the new design included a rubric that outlined specific resources to achieve both short- and long-term goals compared with 33.33% for the current form (P < 0.05).

## Discussion

Self-determination theory (SDT) links learner intrinsic motivation with a sense of competence, autonomy, and relatedness. The self-assessment process is complex and dynamic [3] but serves all three components proposed by the SDT [4].

Resident self-assessment is a process that plays a crucial role in understanding the trainee’s perspective of their strengths and challenges and obtaining key information regarding their insight into their performance. Self-assessment is subjected to the rater effect and commonly requires calibration with faculty feedback, which is essential for a "guided self-assessment" [5] of their competency.

The metric emphasizes a sense of autonomy in what residents find relevant to communicate. It empowers them to choose their own goals, prioritize them, and select the strategies to achieve them. It is an opportunity to personally document their progress towards independence as the final scope of their professional development. 

By aligning their self-evaluation with the information received from external sources (faculty, advisors, peers, and patients), the learners understand the importance of relatedness and experience the connection to others with the scope of becoming a better self. Subsequently, it promotes a sense of belonging and being valued, which is central to the psychological progress of the learner.

Grounded in SDT, we could advance the theory that a quintessential part of designing or redesigning the self-evaluation metric is the learner’s perspective of the tool used. There are a few aspects that are worth exploring. One aspect is the psychological effect on the learner, who considers themselves the center of the activity: not just the recipient of another modality to evaluate their performance but one that respects their opinion and autonomy. Collaborating with the learner on designing a new metric also fosters trust and connection between the residents and the program and increases their relatedness, as proposed by SDT. In addition, the learner’s view brings to light the inherent flaws of a new metric that could remain otherwise obscure to the program.

Based on this concept, we considered resident inclusion in designing the metric as an integral part of the psychological growth of the learners. Residents felt connected with the program and demonstrated once again that the learning process hinges on communication and transparency and that the activities we design with the learners enhance their self-esteem and contribute on a larger scale to their well-being.

Regarding the results of our collaboration, the self-assessment format has changed its configuration. In general, the form should include self-ratings on the ACGME six competency areas, duplicating or modifying the verbiage utilized in the milestones. The ideal number of questions included in a self-assessment remains undetermined. A more extensive questionnaire might compete with the lucrative scope of the activity, which is to focus on a few areas of improvement. A detailed form, although more comprehensive, would also take more time to complete, which is another barrier to finalizing tasks in the contemporary residents’ workflow. The group of residents and faculty envisioned reducing the number of questions by half while keeping them relevant and reflective of the ACGME Milestones 2.0. We decreased the number of items from 60 to 25 for postgraduate year one (PGY1) and PGY2 and 29 for PGY3. The survey results indicated that the majority (80%) of the residents found the current form lengthy and required more time to complete. Per the post-survey results, the proposed structure remained extensive but improved (46.6%). The results demonstrated that a careful examination of the relevant questions is necessary to avoid redundancy and to focus on the opportunities to improve. They also confirmed the need for more awareness of the aim of the self-evaluation, which is to provide an accurate and in-depth assessment of residents' competencies to prevent an under- or overestimation of their performance [5].

The method used for rating is also variable among programs. The ordinal Likert rating scale is most commonly utilized. Still, the semantic differential scale is visually appealing. It adds the benefit of combining the questions with a corresponding binary scale that facilitates an easy selection between the learners' strengths and weaknesses [6]. Our residents and faculty favored the ordinal five-point Likert scale (not confident to very confident) that facilitates responding with a degree of agreement and accommodates the extremes of the scale, inviting learners to identify the competencies that would need immediate attention. 

Besides the specific questions related to the six competencies, the self-assessment tool commonly incorporates an enumeration of the learner's strengths and weaknesses. Our current form reserved a self-appraisal free text section for commenting on their performance in different settings (wards, intensive care unit, ambulatory, and emergency room) and included a rubric where learners listed their strengths and weaknesses alongside strategies and resources that would help improve their performances. The group considered that while the rater should acknowledge their strengths, listing their weaknesses would not guarantee that they would be addressed. As the central purpose of the activity is to be formative and to assist the learner in establishing short-term and career (long-term) goals or objectives, we opted to exclude the opportunity to recite their weaknesses, as that does not imply an action plan. With the help of the mentors, the resident would identify the areas to improve on and prioritize the specific goals necessary to achieve improvement. We used "goals" and "objectives" interchangeably and the I-SMART criteria [6,7] to satisfy specific, measurable, attainable, relevant, and timely objectives. The survey demonstrated that most of our residents agreed that the new form was more helpful in establishing short-term (86.6%) and long-term (73.3%) goals and aligning them with their associated strategies. 

The awareness of the specific resources available to accomplish their goals is a final and critical element of self-evaluation. As part of the current self-evaluation, the opportunity to review the resources was listed last and on the flip page, frequently ignored by the reader. The group of residents suggested that each goal discussed should be followed by the strategies and resources necessary to achieve it. The survey confirmed that 80% of the residents agreed that the new metric could comply with that compared with 33.33% for the current form (P < 0.05).

Structure revision alone may not improve performance rating, and rater training to enhance performance appraisal is effective [8]. Resident training on the metric helps improve the rating accuracy and develops a shared understanding of the scope of the problem [8]. Our program included a training session in resident self-evaluation, but more individualized and comprehensive resident training is beneficial [7].

Formative feedback that complements self-assessment increases the resident's intrinsic motivation [9], is associated with preparedness for self-regulation, is possibly related to enhanced performance [10], and may independently correlate with improved patient care outcomes.

Our study has several limitations. The sample size was small. The survey was anonymous but did not achieve a 100% response rate and used a cross-sectional survey, which may have introduced variability based on training levels.

## Conclusions

To our knowledge, this is the first study that addresses the challenges of designing the self-evaluation tool for IM residents. Resident participation in redesigning a concise, well-structured, visually amiable tool that includes specific, measurable, and timely objectives is a learner-centered pre-requisite that facilitates professional development, increases motivation, increases resident-program relatedness, and enhances programmatic openness and transparency.
